# Clinicians’ perspectives on supporting individuals with severe anorexia nervosa in specialist eating disorder intensive treatment settings

**DOI:** 10.1186/s40337-021-00528-z

**Published:** 2022-01-06

**Authors:** Hannah Webb, Bethan Dalton, Madeleine Irish, Daniela Mercado, Catherine McCombie, Gemma Peachey, Jon Arcelus, Katie Au, Hubertus Himmerich, A. Louise Johnston, Stanimira Lazarova, Tayeem Pathan, Paul Robinson, Janet Treasure, Ulrike Schmidt, Vanessa Lawrence

**Affiliations:** 1 Department of Psychological Medicine, Section of Eating Disorders, Institute of Psychiatry, Psychology & Neuroscience, King’s College London, Institute of Psychiatry, Psychology & Neuroscience, London, SE5 8AF UK; 2 Department of Health Services and Population Research, Institute of Psychiatry, Psychology & Neuroscience, King’s College London, London, UK; 3 South London and Maudsley NHS Foundation Trust, Maudsley Hospital, Denmark Hill, London, SE5 8AZ UK; 4 Institute of Mental Health, University of Nottingham, Jubilee Campus, Triumph Road, Nottingham, NG7 2TU UK; 5 NHS Grampian, Aberdeen, UK; 6 South West London and St George’s Mental Health NHS Trust, London, UK; 7 Surrey and Boarder Partnership NHS Foundation Trust, Surrey, UK; 8 Division of Medicine, University College London, 5 University Street, London, WC1E 6JF UK

**Keywords:** Anorexia nervosa, Clinicians, Healthcare professionals, Eating disorders, Intensive treatment, Qualitative research, Day patient, Inpatient

## Abstract

**Background:**

Admissions to intensive treatment (i.e., inpatient [IP] and/or day patient [DP]) for individuals with severe anorexia nervosa (AN) are common. Growing literature indicates potential risks and benefits of each intensive treatment approach; however, existing research has focused on patient and carer perspectives of these treatments. Also, there is scant empirical evidence available for guiding the parameters of intensive treatments for AN. We therefore explored clinicians’ perspectives and experience of supporting adults with severe AN in intensive settings.

**Methods:**

We conducted twenty one semi-structured interviews with clinicians who deliver intensive treatments (i.e., IP and/or DP) for individuals with severe AN across four specialist Eating Disorder Services in the United Kingdom between May 2020 and June 2021. We asked clinicians about their views and experiences of supporting individuals with severe AN in intensive treatment settings and the challenges and opportunities associated with IP and DP treatment. Data were analysed using reflexive thematic analysis supported by NVivo software.

**Results:**

Five broad and interrelated themes were identified: (1) Intensive Support; (2) The Severity of Patients’ Illnesses; (3) Hope and Recovery; (4) Which Treatment When; (5) Limited Resources; and (6) Carer Burden. We identified various similarities between the two intensive treatment approaches, including the value of intensive and multidisciplinary support and carer involvement, and the challenge of managing complex and unique needs in resource-limited intensive settings. We also found differences in the relationship of treatment to patients’ home environments, the necessity of patient motivation, and the management of risk.

**Conclusions:**

Both intensive treatment settings are valued by clinicians; however, there are unique challenges and opportunities for supporting individuals with severe AN within each. Our findings suggest DP treatment may be used as an alternative to IP treatment for individuals with severe AN. However, clear questions remain over which intensive treatment setting is best suited to which patient when and should be the focus of future research.

**Supplementary Information:**

The online version contains supplementary material available at 10.1186/s40337-021-00528-z.

## Plain English Summary

Some people with anorexia nervosa will need intensive treatment (e.g., inpatient and day patient treatment) during the course of their illness. We interviewed twenty-one clinicians working in Specialist Eating Disorder Services to explore their views on supporting people with severe anorexia nervosa in inpatient and day patient services and about the perceived advantages and disadvantages of these. We analysed the transcripts of these interviews using thematic analysis. We identified similarities between the two intensive treatment approaches. These included the value of intensive and multidisciplinary support, the importance of carer involvement, and the challenge of managing patient’s complex and unique needs in services with limited resources. We also found differences between inpatient and day patient treatment. These included how treatment relates to patients’ home environments, the importance of patient motivation, and managing risk. Day patient treatment may be an alternative to inpatient treatment for people with severe anorexia nervosa. Future research should investigate which intensive treatment setting is best suited to which patient and when.

**Supplementary Information:**

The online version contains supplementary material available at 10.1186/s40337-021-00528-z.

## Introduction

Anorexia nervosa (AN) is a complex mental disorder associated with serious physical and psychological health consequences, impaired quality of life [1, 2], and the highest standardised mortality rates of all psychiatric disorders [3]. Across different healthcare systems (e.g., Europe and USA), intensive treatments (i.e., inpatient [IP] or day patient [DP]/partial hospitalisation) are generally offered in cases of moderate or severe AN, where medical and/or psychological risks are high, or when outpatient [OP] treatment is unsuccessful [4–6]. Approximately one-fifth to one-third of patients with AN require intensive treatments during their illness [7, 8]. Despite well-known ethical, practical, and systemic challenges in providing intensive treatments to individuals with severe AN [9], qualitative research into clinicians’ (often referred to as healthcare professionals) perspectives and experiences of intensive treatments is limited.

Exploring the views of clinicians is of relevance as there is currently a lack of empirical evidence and agreement guiding the parameters of intensive treatments for AN and it remains unclear which treatment setting works best for whom [10, 11]. This is particularly true as factors such as admission criteria, treatment content and goals, and length of stay vary substantially across intensive treatment settings both within and across countries [11]. Furthermore, growing literature indicates potential risks and benefits of each intensive treatment approach [12]. Although IP treatment offers a safe, secure, and accepting environment, it may inadvertently reduce the need for individual responsibility, independence, and autonomy in recovery, and exacerbate certain characteristics associated with AN (e.g., recovery ambivalence, inflexibility) [12]. While DP treatment has been put forwards as an alternative to IP treatment, it tends to be used for more moderate cases [11]. DP settings may permit patients to transfer skills learnt in treatment more easily to life outside, maintain greater social networks and other commitments, be perceived as less coercive, and reduce overall healthcare system costs [10]. However, DP settings may pose challenges relating to management of risk, control of eating disorder (ED) behaviours outside of treatment, carer burden, and pose practical difficulties for those living at a distance from services. As such, conducting qualitative research with clinicians who have experience of managing severe AN in intensive treatment settings gives voice to this group and facilitates a better understanding of a poorly understood and complex area of healthcare [13, 14]. The present qualitative study, therefore, aims to explore clinicians’ perspectives and experience of supporting adults with severe AN in intensive treatment settings and the opportunities and challenges associated with these.

## Methods

Ethical approval was granted by the Wales Research Ethics Committee 5 (Reference: 20/WA/0072).

### Participants

Data for this study were collected as part of the process evaluation for the DAISIES trial [15]; an ongoing two-arm multi-centre open-label parallel group non-inferiority randomised controlled trial evaluating the clinical and cost-effectiveness of two intensive treatment approaches (IP treatment-as-usual versus a stepped-care DP treatment approach [DP treatment with the option of an initial IP stay for medical stabilisation]) for adults with severe AN (defined as AN with BMI ≤ 16.0 kg/m^2^) in the United Kingdom (UK). Participants represented a purposive sample that sought variation in terms of professional background, experience in EDs, and setting, from selected specialist ED Services involved in DAISIES, although participation was informed by staff availability and interest. Clinicians working in these services delivering intensive treatment (IP and/or DP) for people with severe AN were invited to participate via email by the DAISIES Trial Co-ordinator (author BD). We interviewed twenty-one clinicians across four services. Eight clinicians worked only in DP settings, four in IP settings, and two in OP settings, with a further five clinicians working across both OP and DP settings and two clinicians working across both IP and DP settings. The majority of participants (*n* = 15) were from one ED service (South London and Maudsley NHS Foundation Trust), with the remaining (*n* = 6) from the other three sites (South West London and St. George’s Mental Health NHS Trust; Surrey and Borders Partnership NHS Foundation Trust; NHS Grampian). Participants were from a range of professional backgrounds and had varying years of experience in different ED settings (see Table 1). Recruitment continued until the sample was judged to hold adequate information power [16]. This was influenced by our relatively broad aim, interest in exploring the diversity of opinions and exploratory cross-case approach to analysis.

**Table 1:** Setting, role and years of experience in eating disorders for each participant

Participant ID with work setting	Role	Years of experience in EDs
P1-OP	Consultant Psychiatrist	10+
P2-IP/DP	Consultant Psychiatrist	5–10
P3-IP	Consultant Psychiatrist	10+
P4-DP	Occupational Therapist	5–10
P5-OP/DP	Consultant Psychiatrist	10+
P6-OP	Clinical Service Manager	10+
P7-DP	Nurse Specialist	0–5
P8-IP	Consultant Psychiatrist	0–5
P9-DP/IP	Nurse Therapist	10+
P10-OP/DP	Dietician	10+
P11-OP/DP	Nurse Specialist	0–5
P12-IP	Counselling Psychologist	Not reported
P13-DP	Mental Health Nurse	0–5
P14-DP	Assistant Psychologist	0–5
P15-IP	Counselling Psychologist	10+
P16-DP	Assistant Psychologist	0–5
P17-DP	Mental Health Nurse	0–5
P18-OP/DP	Occupational Therapist	0–5
P19-DP	Occupational Therapist	0–5
P20-OP/DP	Consultant Psychiatrist	10+
P21-DP	Day Unit Manager	10+

*OP* outpatient, *DP* day patient, *IP* inpatient, *ED* eating disorder

### Data collection

All participants provided informed consent. We conducted semi-structured interviews via Microsoft Teams, at a time convenient for the participant. Interviews were carried out by researchers supporting the DAISIES trial (BD, DM, GP, and MI) between May 2020 and June 2021. All interviews were audio-recorded and transcribed verbatim, with identifiable information removed.

The topic guide (see Additional file 1) was designed by authors MI, US, VL and BD and used open-ended questions to explore participants’ views and experiences of supporting individuals with severe AN in intensive treatment settings, the advantages and disadvantages of the two different intensive treatment approaches, and their thoughts on implementing the DAISIES trial within their sites. In addition, due to the COVID-19 pandemic (as declared by the World Health Organisation on 11th March 2020), we included further questions to explore clinicians’ experiences of the impact of the pandemic on treatment delivery and supporting individuals with severe AN [17]. For the purpose of this study, only data relating to supporting individuals with severe AN in intensive settings prior to COVID-19 are reported.

### Data analysis

We used reflexive thematic analysis (supported by NVivo 12) for identifying, analysing, and interpreting data-driven themes, following the six phases outlined by Braun and Clarke [18–20]. We took an inductive approach, grounded in interpretivism, that acknowledged the importance of the participants’ and the researchers’ interpretations and understanding of the phenomenon under study [18–20]. First, transcripts were read and reread to ensure data familiarisation. Three transcripts were then coded by two researchers (DM and VL) to help us interrogate the data and elucidate alternative interpretations. Through collaborative discussions, initial codes were sorted into preliminary themes, ensuring that early interpretations and ideas about patterns in the data were grounded in the participants’ accounts. A third researcher (HW) then reviewed the coding framework and, through ongoing discussions (with VL) and an iterative process of refining and defining themes, developed the final coding framework: ensuring data cohered, no codes were ignored, and all perspectives were represented. HW (mental health researcher, practitioner and former service user) and VL (social scientist) drew upon prior experience of conducting applied qualitative research in EDs and other areas of mental health. HW also drew upon her knowledge and experience of ED treatment and reflected throughout on how this might influence her approach to the data. The first author (HW) then summarised the analysis into a written report which was circulated and approved by all the authors. For each theme, direct supporting quotations are provided, anonymised using participant numbers suffixed with participants’ current work setting (i.e., OP, DP and/or IP).

## Results

**Fig. 1:**
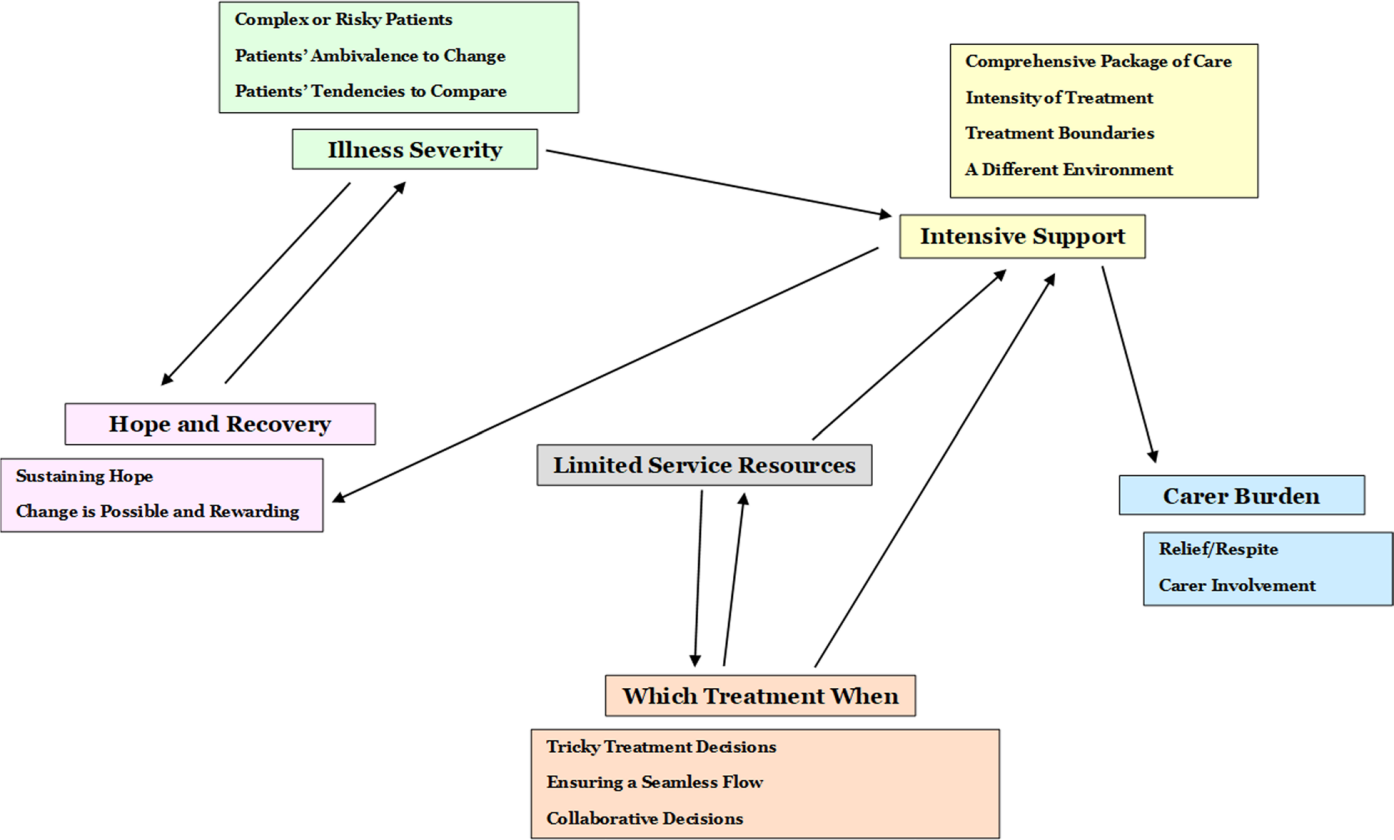
Thematic map

Five themes were generated from the data (see Fig. 1 for a thematic map): Intensive Support; The Severity of Patients’ Illnesses; Hope and Recovery; Which Treatment When; Limited Resources; and Carer Burden.

### Theme 1: intensive support

#### Comprehensive package of care

Many clinicians valued the *“multidisciplinary approach”* (P15-IP) and “*menu of therapy options*” (P2-IP/DP) available in both intensive settings, which includes meal and nutritional support, physical health monitoring, occupational therapy, nursing and consultant input, and psychology (e.g., cognitive/dialectical behavioural, family, and group therapy). Group work forms an integral part of DP programs.

Several clinicians described simultaneously supporting patients with the physical and psychological consequences of severe AN, although one clinician raised concern over how IP treatment can separate the mind and body.

It becomes too much about giving medical interventions like physical obs, NG feeding, blood tests… it's become so disconnected (P12-IP)

However, many noted the merit of being in hospital environments to ensure robust medical monitoring. Some IP clinicians specifically valued being able to provide nutritional supplements or nasogastric feeding. Nonetheless, two clinicians found nasogastric feeding personally challenging.

I understand the need for it, and sometimes it does feel like the only thing that’s actually going to stop a patient from dying. But again, particularly if it’s being delivered under restraint, that can be tough to sit with (P12-IP)

#### Intensity of treatment

Clinicians in both settings described IP treatment as the highest level of support, with several specifically referring to the contained, structured, 24/7 care, which encourages patients to focus on recovery and let go of other responsibilities and enables clinicians to develop thorough formulations.

Giving that full 24/7 support to really push for recovery in a very concentrated way. That's not to be underestimated (P1-OP)

Several clinicians mentioned the mortality associated with severe AN, recognising that IP intensity is often necessary.

Potentially, it’s a life-saving measure (P20-OP/DP)

At the same time, the majority of clinicians raised concerns over the risk of IP institutionalisation, particularly for longer admissions. Clinicians described how patients can *“become reliant”* (P3-IP) or *“quite passive”* (P5-OP/DP), as the ward becomes a safe and containing environment.

DP clinicians also described their services as intensive, although emphasis was placed on the *“terrific structure”* (P10-OP/DP), with Monday-to-Friday programs being likened to full-time education or work. Program hours were similar across sites (ranging between 8 a.m. and 4 p.m.), with one site offering optional evening support. Patients had to commit to core times/days, but it was recognised that each patient’s needs, and commitments, differed. Emphasis was placed on an *“individualised approach”* (P4-DP).

Time outside of DP treatment was regarded both positively and negatively. Patients are supported to take *“some responsibility”* (P9-DP/IP) and “*participate in a much more active way”* (P5-OP/DP) in their treatment. Several DP clinicians mentioned patients’ motivation and willingness to change, and one articulated that DPs have been *“sort of cherry picked as those who have, are motivated”* (P10-OP/DP). Conversely, many clinicians suggested a challenge of DP treatment is managing the hours that patients are not in intensive treatment. Clinicians raised concerns over external distractions (e.g., childcare, work) and “*patients compensating for all the good work”* (P15-IP) by pushing ED behaviours into evenings or weekends. Several recognised that DPs’ progress may be slower than IPs’.

Clinicians in both settings valued their ability to really get to know patients due to the lengthier nature of intensive treatment. *“You can get to know somebody as a person”* (P4-DP) and form a *“warm [therapeutic] relationship”* (P12-IP). Learning what is important to each patient and getting to know their unique journey was seen as particularly valuable.

Clinicians also valued having time to support patients to practice and develop skills (e.g., grocery shopping, cooking and canteen groups, through food exposure tasks).

It was really practical interventions we were doing. I think you can talk and talk but it doesn’t mean you're going to do something about it. I guess by practising it can help them increase their confidence (P19-DP)

This concept was prominent among DP clinicians and several mentioned using a phased approach, moving patients from having *“less of the control”* to taking *“back the control in an appropriate way”* (P21-DP). In contrast, only two IP clinicians described a phased approach, in which patients practice new skills during day or overnight leave. One clinician recognised that many patients initially struggle.

Not all patients can immediately, kind of, manage. Some of them, they have setbacks, we're able to be here to, kind of, support them (P8-IP)

#### Treatment boundaries

Although not mentioned by IP clinicians, recurrent among DP clinician narratives were descriptions around treatment boundaries. Clinicians described weight gain targets and expectations around attendance and completion of meals and snacks. If patients consistently struggle to meet the boundaries, they may be asked to take a week out. After two weeks out, patients are discharged (stepped up/down). One clinician explained that asking patients to take a week off was *“obviously a really uncomfortable conversation… [patients may feel] they’ve failed recovery… or they’re being punished”* (P18-OP/DP). Indeed, clinicians recognised that the boundaries may not suit everyone.

Some services offered two DP programmes to accommodate different levels of boundaries. One *“geared towards patients who are signing up for full recovery”* (P8-IP), with *“stricter boundaries”* (P14-DP), and another for patients who may be ambivalent about clinical recovery but may want *“a better quality of life”* (P19-DP) or *“short intervention goals”* (P21-DP).

#### A different environment

IP treatment was regarded as a complete removal from patients’ home environment. Two IP clinicians described how removal from potentially problematic home environments can be beneficial. However, several others suggested this disruption can be challenging. It can *“add additional pressure, financially or academically”* (P8-IP) and can exacerbate isolation due to being away from family/friends, particularly for national patients who experience increased geographical barriers. Indeed, many raised concerns about IP treatment being a *“little bubble”* (P7-DP).

It kind of cuts you off, creates a barrier between you and the rest of the world (P6-OP)

In contrast, DP treatment was regarded as more applicable and supportive of the transfer of skills and learnings across settings. Patients can *“generalise their treatment a bit more easily”* (P2-IP/DP), better maintain social connections, and fit treatment around other commitments, due to returning home each evening/weekend.

It then makes a less of a jump to… move away from their eating disorder and back into life (P14-DP)

### Theme 2: illness severity

#### Complex or risky patients

Clinicians described challenges relating to the complexity of severe AN.

Many IP clinicians described illness consequences (such as cognitive and social difficulties) and their impact on patient engagement. For example, two clinicians mentioned difficulties establishing trusting relationships and another described how severe AN *“gets in the way of ordinary decision making”* (P15-IP). There was consensus that it takes patients several weeks to adjust to IP wards—*“going at the patient’s pace is really important”* (P12-IP). Similarly, a DP clinician acknowledged the challenge of working with patients with long-standing illness.

They're often more stuck… than some of the patients newer to the illness… and they're much more challenging to work with in that way (P10-OP/DP)

Prevalent among DP clinicians’ narratives was concern over managing risky patients in less intensive (than IP) settings. Concerns were raised over a *“complexity around their presentation”* (P4-DP) e.g., medical risks associated with low weight and psychiatric comorbidities. More seriously unwell patients who need *“more eyes and more treatment”* (P17-DP) were particularly troubling.

There's an anxiety there about holding that [risk] responsibility sometimes (P4-DP)

#### Patients’ ambivalence to change

Related to the complexity of severe AN was patient ambivalence and perceived resistance towards treatment.

IP clinicians described how severe AN is commonly *“so entrenched”* (P3-IP) that patients may be unwilling to be admitted to intensive treatment, struggle to engage, or relapse after discharge. IPs resistance to change and high ambivalence were frequently mentioned.

DP clinicians described how patients’ ambivalence to change affects treatment engagement. It was recognised that DP services depend significantly on high motivation to attend daily and compared to IP treatment, DP puts greater responsibility on the individual. Whilst patient responsibility was largely regarded positively, it could be problematic for some.

It puts a lot more responsibility on the individual to take… charge of their recovery and treatment and be motivated to… we can't force people to come in (P14-DP)

Several DP clinicians suggested some patients see DP treatment as a way of avoiding an IP admission. This leads to frustrations when patients are not fully committed.

#### Patients’ tendencies to compare

Concerns arose over patients’ tendencies to engage in comparisons and negatively influence each other. The majority of clinicians (including DP clinicians) raised concerns over potentially harmful effects of IP environments, whereas only a few clinicians raised concerns over DP environments.

Being in an IP environment 24/7 was described as potentially triggering and distressing, although clinicians across both settings described the possibly harmful impact of a *“negative culture”* (P1-OP) (e.g., if recovery is regarded as undesirable).

There’s perfectionism and… a lot of competition… then they'll see someone who they think is getting away with the behaviour that they're trying very hard not to engage in, that can be very difficult (P12-IP)

Particular thought was given to the impact of being around others who are really struggling or very low weight.

Sometimes patients do pick up bad behaviours… so in that respect… the outcome from the admission may not be all positives (P20-OP/DP)

In both settings, concerns were raised over patients newer to the illness.

Sometimes they haven't come across other people with eating disorders before… that can be supportive in some ways, they can also pick up some dreadful habits (P10-OP/DP)

Contrastingly, several clinicians described the positives of peer-to-peer support, validation and understanding. Clinicians described how patients can witness others facing their difficulties, *“exchange their experiences”* (P3-IP) and form *“good supports and friendships”* (P1-OP), particularly when the culture is recovery oriented.

### Theme 3: hope and recovery

#### Sustaining hope

Several clinicians mentioned the importance and challenge of sustaining hope (both patients’ and their own) that recovery is possible.

We are a little bit of a microcosm of the kind of the sickest of the sick, at their most sick. So, trying to sustain hope and remembering that recovery is possible… for the patients and the staff group can be quite a challenge (P15-IP)

Moreover, clinicians described the challenge of holding onto hope when supporting those who have *“been through a lot of treatment models”* (P21-DP) or who experience treatment as *“something horrible being done to them”* (P15-IP).

#### Change is possible and rewarding

Clinicians in both settings valued supporting and witnessing patients make physical, cognitive, and psychological changes and/or fully recover. Emphasis was placed on *“great numbers and full-blown recovery”* (P6-OP) as well as on small changes (e.g., a patient being able to attend a wedding). Many also appreciated supporting patients to *“work towards their own definition of recovery”* (P17-DP).

Their thinking, personality and emotions wake up again, and although that's painful for them, it's just so lovely to see people reappear (P15-IP)

### Theme 4: which treatment when

#### Tricky treatment decisions

Deciding which treatment is most appropriate at which point was described as challenging. Generally, the decision to admit patients to intensive treatment depended on the severity of the patient’s illness and involved physical and mental health considerations. Clinicians commonly first considered DP and then IP treatment, although it was recognised that each patient’s journey is unique.

Our patients are complex and individual human beings and there's always going to be a need for different types of interventions for different types of patients with different types of history (P15-IP)

A few clinicians explained that their services have no specific protocols or tools to facilitate decision-making around admissions to intensive treatment and another described uncertainty around the decision.

Some decisions are quite intuitive, that are made by the clinicians, and we don't know whether these decisions are good or bad (P3-IP)

Decision-making arose as particularly challenging when considering newly referred patients (i.e., should they be admitted immediately?) and risky patients with longer illness durations.

The people who are just stuck, who are sort of managing their life, but actually by not offering something more intensive we've consigned them to a life of anorexia (P1-OP)

Two clinicians mentioned the challenge of holding risky patients as OPs and several others acknowledged that patients may deteriorate quickly but often do not want intensive treatment.

#### Ensuring a seamless flow

Clinicians largely described patients’ movements between OP, DP and IP treatment as difficult. It was recognised that there are substantial differences between levels of care in IP and OP settings, yet individuals often go straight from one to the other. Hospitalisation was described as overwhelming and distressing due to the sudden loss of freedom and control.

I do wonder whether that smaller transition might be easier to manage (P8-IP)

IP clinicians also recognised the difficulty of patients having to quickly transfer learnings from the ward to their home. Although some patients are offered a stepped-care approach (stepping-down from IP to DP), many often wish to be discharged straight into the community.

In the past there was more time for people to… do the discharge process very gradually, nowadays it's done a bit more quickly and so that's also then a bit of a shock to the system (P1-OP)

DP clinicians described the challenge of different ED services feeling *“very, like, separate”* (P16-DP) for patients and clinicians and a desire for better communication between services. One DP clinician considered how some patients transitioning from IP to DP may *“feel like they’re stepping backwards”* (P18-OP/DP) due to a reduction in freedom and control, compared to that afforded at the end of their IP admission (e.g., due to extended leave), whilst another described the importance of *“a crossover period…”* (P21-DP). This clinician also emphasised the importance of a phased, planned discharge from DP treatment.

#### Collaborative decisions

Almost half of the clinicians emphasised that intensive treatment is a collaborative decision based on shared understanding, the patient’s choice, and families/carers if permitted.

We’d involve the family in the decision making as well, because often the patient themselves can't see how unwell they are (P1-OP)

Additionally, two clinicians mentioned that on occasions they have to use the Mental Health Act but that this is *“quite rare”* (P5-OP/DP). One clinician described being aware of the *“power balance”* (P15-IP) in IP services.

It can cause a lot of tension in the [therapeutic] relationship… we can really impose things on people… we try to be acutely aware of that, but even with a lot of work you can't necessarily get patients on board with seeing things the same way that you can (P15-IP)

### Theme 5: carer burden

#### Relief/respite

Clinicians in both settings suggested intensive treatment is helpful for carers.

IP treatment was frequently described as providing carers relief and respite. Nonetheless, a few clinicians suggested that reduced carer responsibility can be harmful. Family members struggle with *“not getting the… full picture”* (P21-DP) and feel unprepared when the patient returns home.

DP treatment also offered partial periods of relief and respite and reduced carer responsibility. Described as *“a bridge”* (P9-DP/IP) between IPs and OPs, DP treatment can leave some carers with *“stress and anxiety”* (P17-DP), particularly if the patient is very unwell.

The burden on carers is much higher than if you have proper inpatient treatment (P1-OP)

#### Carer involvement

Clinicians felt intensive treatment provides greater opportunity for family involvement e.g., family therapy, carer workshops, frequent communication and involvement (e.g., practicing skills, sharing care plans). In DP treatment, emphasis was placed on carers taking an *“active part from the start”* (P5-OP/DP) due to patients’ ongoing connection to home.

With the family therapy and all the carers support, we are bringing the family closer together as well and, kind of, empowering them to support the patients (P8-IP)

Nonetheless, a minority of clinicians described concerns over insufficient carer support.

One IP clinician suggested their service was lacking a carers’ support group and described family work as *“hit and miss”* (P6-OP). Several others suggested carers may experience *“anxieties around separation”* (P11-OP/DP), particularly those whose loved ones do not consent to open communication between clinician(s) and carer(s). Moreover, one clinician specifically described the challenge of inadequate/inappropriate carer support:

You need to get the carers on board to make changes within the family, otherwise the individual changes but the carers don't, and then there's a mismatch when the patient goes home (P6-OP)

Additionally, several DP clinicians described how they occasionally hear from carers who are *“upset”* (P16-DP) or *“angry”* (P18-OP/DP) regarding their loved one’s treatment, in part due to not understanding treatment plans, boundaries or clinical reasonings.

### Theme 6: limited service resources

Underpinning the majority of clinicians narratives were concerns over limited resources. Clinicians valued being part of a *“diverse team with diverse skills”* (P12-IP) and team members were a valuable resource, with reflective groups, clinical supervision, and management rounds all facilitating a supportive staff culture. However, in both settings, clinicians mentioned inadequate numbers of (specialist) staff. Due to this, one clinician described how patients are sometimes *“waiting for key interventions”* (P21-DP).

More funding for more staff would be the best to be able to provide more care for more patients (P17-DP)

Managing patients with differing diagnoses, body mass indexes (BMIs), meal plans, and triggers was described as difficult within these resource-limited settings.

Clinicians in both settings also described the challenge of limited capacity and lengthy waiting lists. IP wards *“rarely have empty beds”* (P15-IP), leading to service provision challenges and difficult treatment decisions e.g., a London IP service had needed to send patients to Scotland. Similarly, DP clinicians described waiting list pressures and capacity limitations.

In IP services, particular concerns were raised around changes to admission aims. Several clinicians expressed concern over the *“emphasis in feed up and out”* (P6-OP) and pressure of shortening admissions.

A few years ago, we said you should treat them to a BMI of nineteen when they are inpatients, but at the moment we only treat them up to a BMI of sometimes only fifteen or so and discharge them (P3-IP)

Additionally, several DP clinicians raised concerns over equity of access in the face of limited resources. Two clinicians, working across rural and urban areas, felt that day treatment is *“not an equitable service”* (P2-IP/DP) i.e., only patients who live within a certain proximity or have sufficient finances for travel can attend. Two other clinicians voiced concerns over how BMI criteria may limit access: *“do you have to achieve a really low BMI to get help?”* (P15-IP). Another explained how sometimes those who seemed sickest may be *“jumped up the waiting list”* (P21-DP).

## Discussion

We explored clinicians' perspectives and experiences of managing severe AN within intensive treatment settings, with a particular focus on the relative merits and drawbacks of each approach. In IP and DP settings, similar opportunities (e.g., value of intensive multidisciplinary support; change and recovery; carer involvement) and challenges (e.g., decision-making around intensive treatments; supporting individuals with complex and severe illnesses within resource-limited settings) were described, complmenting existing research into patients’ and (albeit more limited) carers’ perspectives and experiences of intensive treatment [21–25]. Differences were also evident between IP and DP settings. Despite difficulties in managing patients’ ED behaviours outside of treatment and the necessity of patient motivation, DP treatment appears to permit greater links to one’s home environment, greater responsibility in recovery, and a smoother transition out of intensive treatment. These findings provide support for DP treatment as an alternative to IP treatment for individuals with severe AN [10, 26] and calls for the introduction of more evidence-based intensive DP treatment programs to meet the needs of all patients with AN [27]. Further research comparing the two intensive treatment approaches is necessary, as while both settings are valued, it remains unknown which treatments work best for whom at what time.

The intense and multidisciplinary provision of care, recognised as crucial for individuals with AN [6, 28], was valued across intensive settings. Clinicians highlighted the importance of consistent nutritional support and medical monitoring (particularly within IP settings), and offering various types of family and psychological support, even for individuals who are severely medically compromised. However, within IP settings, there were some concerns of an emphasis of physical, over psychological, recovery. This is consistent with patients’ perspectives of a need to focus more on psychosocial factors [23, 29]. Additionally, clinicians felt that the wrap-around nature of intensive settings supported them to challenge patients’ disordered thoughts and behaviours through frequent and graded practical (food-related) skills groups and exposure tasks. DP clinicians tended to report a strong focus on these, whereas IP clinicians sometimes felt time and resources for these activities were limited. Indeed, they are integral elements of AN treatment and are valued not only by clinicians, but also patients [25], as they support the transfer of skills to life outside of treatment, which is an important basis for sustainable recovery [30, 31]. Overall, these findings highlight the value of a comprehensive and multidisciplinary package of intensive treatment.

Differences arose between settings regarding their relationship to patients’ home environments and in supporting patients' autonomy/responsibility in recovery. In this study we found a clear concern over the risk of IP institutionalisation. Clinicians felt that IP treatment provides some patients with a safe, supportive and accepting environment, and the opportunity to fully focus on recovery. However, for others, they felt it can lead to a loss of autonomy and personal responsibility in recovery, negative peer comparisons and influences, and social/occupational isolation. In contrast, clinicians felt that DP treatment facilitates greater links to patients’ home environments (and consequently, increased opportunities to work/volunteer, and maintain social relationships), greater autonomy/responsibility in recovery, and potentially smoother transitions out of intensive treatment. Yet DP clinicians also raised concerns over patients’ engagement in ED behaviours outside of treatment hours, patients’ being unable to sufficiently focus on recovery, and (lesser) concerns over negative peer comparisons and influences. These juxtapositions of supportive, yet potentially harmful/problematic intensive environments concur with previous research [12, 32–34], including that into IPs’ perspectives [21, 35, 36]. Crucially, these findings highlight the importance of an individualised approach, as whilst one environment may be preferred and/or effective for one individual, it may be disliked and/or harmful to another. Further research is required to determine who may benefit from which setting.

This was a clear challenge across both settings. IP and DP clinicians found it difficult to know which setting works best for whom at what time, especially for patients newer to ED services or those with longer illness duration. Moreover, clinicians recognised transitions into and out of intensive treatments as difficult (for patients, carers and clinicians)—indeed, increasing evidence encourages a planned and phrased transition [37, 38]. Whilst clinicians advocated for shared decision-making with patients, families and clinicians to support transitions between settings, balancing beneficence with the patients’ preferences can be challenging [39]. Clinicians described a lack of guidance and protocols for decision-making around intensive treatment, which is a longstanding area of uncertainty [31]. There is scant empirical research comparing the two intensive treatment approaches, with the majority of studies being small and uncontrolled, as outlined in [15]. The largest (*n* = 172) and most rigorous study to date, although done in adolescents, showed that DP treatment (using a stepped-care approach: a brief IP stay prior to ‘stepping-down’ to DP treatment) was non-inferior to IP treatment in terms of clinical effectiveness [10]. Given the limited evidence, we are currently conducting the DAISIES trial, a non-inferiority randomised controlled trial comparing IP treatment to a stepped-care DP approach for adults with severe AN [15]. We hope findings from this trial will expand treatment options for severe AN and increase our understanding of what works best for who.

Despite research and media highlighting the longevity and complexity of AN, recovery is possible [40]. Clinicians valued seeing patients’ improvements throughout intensive treatment and emphasised the importance of sustaining their own, and patients’ hope that recovery is possible [24, 41]. A supportive and collaborative team are considered to be crucial [42]. Indeed, consistent with a growing body of research [43–45], the importance of an individualised and patient-centred approach to treatment and recovery was highlighted, whilst noting the ethical considerations of managing patients’ desires and ambivalence towards recovery, within the context of limited resources [39]. It was recognised that DP programs may limit their offer to a select group of patients who are motivated and able to adhere to treatment boundaries. While boundaries are important (e.g., as DP places greater responsibility on the patient and ambivalence to recovery in AN is common; [31]), this may limit access for certain patients who might benefit from intensive support. Interestingly, in recent years, a harm reduction approach in EDs has been proposed for those not ready for or currently able to pursue recovery [46]. Whilst contentious, this model may provide an opportunity for a safe and supportive environment, focusing primarily on reducing risk, maintenance, and improving quality of life [47]. This approach was reflected in two DP programs described by clinicians, which have less rigid treatment boundaries and a greater emphasis on quality of life. Taken together, these findings highlight the importance of an individualised approach to recovery, and in particular, the need to consider how treatment boundaries may impact some patients’ ability to access intensive treatment suited to their preferences, needs and recovery trajectories.

There is growing evidence that carer support is valuable, even for adults with AN, in preventing relapse and sustaining recovery [8, 48]. Whilst clinicians recognised the importance of this, they identified a contradiction in the impact of intensive treatment on carers. Echoing carers’ perspectives [49], IP and DP treatment offer carer relief and respite and for some, increased opportunities for carer involvement (as compared to OPs), both of which may decrease carer burden and distress [50]. However, depending on services’ resources and patients’ preferences, intensive treatment can lead some carers to feel isolated from their loved one’s care and unprepared for their transition back to the community/home, particularly if support for carers is lacking [49]. Due to the nature of DP treatment (i.e., patients are home at evenings/weekends), this likely facilitates an easier transition back to full-time home life for patients and their carers. These findings highlight the need to continue and enhance provisions for carers at all stages of intensive treatment [50, 51].

A common factor underlying all themes was the lack of resources in specialist ED services—they are overburdened and under-resourced [25, 52, 53], which increases waiting lists and duration of untreated ED, and consequently, leads to poorer outcomes [54, 55]. Indeed, limited resources for ED care and treatment have been described internationally [56] and indicated by clinicians in previous research [42]. Within the resource-limited contexts, managing the severity, complexity and diversity of patients’ illnesses arose as a clear challenge, echoing previous research into clinicians’ perspectives [9, 41]. For example, psychiatric comorbidities, as well as characteristics such as resistance to change and denial of illness severity, are common in EDs [1], adding complexities and considerations in intensive treatment. Moreover, despite concerns over institutionalisation, clinicians described apprehensions over having to discharge IPs at potentially still compromised BMIs (due to demand for, and pressure on, beds). Discharge BMI is an important predictor of medium- and long-term outcomes [57]. It may be that patients are being admitted at increasingly low BMIs, yet time is too pressurised to support a full medical (and psychological) recovery. These findings further emphasise that investment is vitally needed to address demand and capacity in adult ED services, particularly in light of the COVID-19 pandemic [58]. An individualised, patient-centred approach is only possible if resources are significantly increased.

## Strengths and limitations

This is the first study to consider clinicians’ perspectives and experiences of intensive settings, with a focus on severe AN, adopting a strengths/opportunities and deficits/challenges approach. There was diversity in the DP offerings across sites, consistent with findings in the literature [8], which enabled us to examine the value of different approaches. However, all four sites had a DP service, which is not representative of the UK more broadly (< 40% of UK ED services provide intensive DP treatment) [27]. Clinicians from various professional backgrounds with differing lengths of experience in EDs participated, allowing a wide range of perspectives to be considered. However, the majority of clinicians interviewed were from one London service, and the UK-focus of this study may limit the transferability of these findings due to global variations in ED treatment provision and pathways [11]. Additionally, the research process (interviews and analysis) was performed by several different researchers, due to resource provisions. Nonetheless, our approach was underpinned by best practice guidelines for thematic analysis [18], and we made efforts to adhere to guiding principles of transparency, reflexivity and integrity, e.g., through ongoing researcher reflexivity; rich descriptive data; a detailed methodology [59, 60].

## Conclusions and implications

This study provides insight into clinicians’ perspectives of supporting individuals with severe AN in intensive treatment settings. Our findings expand upon previous studies exploring topics such as the value of intensive and multidisciplinary support, the importance of carer involvement and support, and the challenge of managing complex and unique needs in resource-limited intensive settings. We recommend that urgent investment is needed to support over-burdened and under-resourced ED services.

Our findings emphasise the importance of an individualised and patient-centred approach to the treatment of and recovery from severe AN. It also highlights the need for further research into patients’ and carers’ views and experiences of intensive treatments for severe AN, and the processes of collaborative decision-making and transitions around these treatments from the perspective of clinicians, patients and carers. We also suggest that services continue to develop and invest in opportunities for carer involvement in intensive ED treatment, ensuring they are fully supported throughout this process. Our study also illustrated various differences that exist between the two intensive treatment approaches, which present unique challenges and opportunities within each setting. Our findings suggest that DP treatment may, for some, be an acceptable alternative to IP treatment, potentially permitting greater treatment accessibility, carer involvement and likely, cost savings. Therefore, clinicians need to consider this when offering intensive treatment to patients with severe AN.

While both intensive treatment settings are valued, clear questions remain over which intensive treatment setting is best suited to which patient when, emphasising the timely nature of the DAISIES trial. Ultimately, in line with a recent report, our findings support the need for investment, innovation and research into different models of intensive treatments for individuals with EDs [61].

## Supplementary Information

**Additional file 1:** Topic Guide.

## Data Availability

The datasets generated and/or analysed during the current study are not publicly available but are available from the corresponding author on reasonable request.

## Abbreviations

AN: Anorexia nervosa
BMI: Body mass index
DP: Day patient
ED: Eating disorder
GP: General practitioner
IP: Inpatient
OP: Outpatient
NHS: National Health Service
UK: United Kingdom
