# Supplementary material for: Clinicians’ perspectives on supporting individuals with severe anorexia nervosa in specialist eating disorder intensive treatment settings
Source: J Eat Disord. 2022 Jan 6;10:3. doi: 10.1186/s40337-021-00528-z (PMC8733908; doi:10.1186/s40337-021-00528-z)
Supplement: Supplementary file 1 — Additional file 1: Topic Guide. [file 40337_2021_528_MOESM1_ESM.docx]

**Clinicians’ perspectives on supporting individuals with severe anorexia nervosa in specialist eating disorder intensive treatment settings**

Authors: Hannah Webb, Bethan Dalton1, Madeleine Irish, Daniela Mercado, Catherine McCombie, Gemma Peachey, Jon Arcelus, Katie Au, Hubertus Himmerich, A. Louise Johnston, Stanimira Lazarova, Tayeem Pathan, Paul Robinson, Janet Treasure, Ulrike Schmidt, Vanessa Lawrence

**SUPPLEMENTARY FILE**

**SUPPLEMENTARY A. TOPIC GUIDE**

The topic guide used in the current study is shown below. Please note, only data collected in Part 1 of the topic guide was analysed for the purposes of the current study. Data collected in the remaining parts of the topic guide will be presented elsewhere.

**Part I.**

To begin with, I just want to get an idea of the setting you currently work in. In answering these first set of questions, please think about the time before the current COVID-19 situation started:

- Can you tell me about the setting that you work in and how you normally support people with severe AN?
- For you, what are the challenges of providing care to patients with severe anorexia nervosa in this setting?
  - *For those working in outpatient settings:* Could you tell me a bit about the decision making around admissions to either inpatient of day patient treatment and how you approach that with your patients?
  - *For those working in inpatient/day patient settings:* Could you tell me a bit about stepping patients up or down between inpatient, day patient and outpatient treatment?
- For you, what are the most rewarding aspects of providing care to patients with severe anorexia nervosa within this setting?

Thank you. We would also like to hear about your views around [day patient/inpatient] treatment as usually delivered in your service, and what the impact of receiving treatment in these settings is for patients and families:

- What do you believe are the positives of [day patient/inpatient] treatment for patients with severe anorexia nervosa and their carers?
- What do you believe are the negatives of [day patient/inpatient] treatment for patients with severe anorexia nervosa and their carers?

**Part II.**

Thank you. Given the current coronavirus pandemic, we also wanted to hear a bit about how this ongoing crisis is impacting /has impacted your work with severely ill anorexia nervosa patients.

- - How are you managing these severely ill patients currently?
  - How is your [inpatient/day patient/outpatient] service running during this time?
  - What is the impact of the current crisis and the way your services are running on these patients and their families?
  - How do you expect the current crisis will affect your [inpatient/day patient/outpatient] service in both the shorter- and longer-term future?

**Part III.**

The last set of questions is more specific to the DAISIES trial. This information will help us implement the study and optimise recruitment.

To give a bit more detail - the DAISIES trial is a randomised controlled trial whereby patients with severe anorexia nervosa will be allocated by chance to either stepped care day patient treatment or inpatient treatment. We will test whether the stepped care treatment approach is as good as inpatient treatment for a number of clinical outcomes, including BMI, eating disorder symptoms and social functioning. In addition, we will look at the cost of the two different treatment approaches and explore whether there are differences between them in terms of their value for money.

Inpatient treatment-as-usual is currently the standard care pathway for patients with severe anorexia nervosa. In this care pathway, patients are admitted to specialist eating disorder inpatient units and are treated by a multidisciplinary team. The stepped-care day patient approach combines intensive day patient treatment with the option of inpatient treatment for medical stabilisation and progression to day patient treatment at the earliest opportunity. Decisions around stepping down from inpatient to day patient treatment will be based on regular risk assessments which will start when patients are assessed for study eligibility. In both of these treatment pathways, the aim would be to treat patients until they reach a healthy weight and normalise their eating or get as close to this point as possible (for example, a previously agreed BMI). Do you have any questions about these two intensive treatment approaches?

The patients we will include in the trial are those who have severe anorexia nervosa, this includes all those who would usually receive either day- or inpatient treatment, but in addition also those patients who have not improved with outpatient treatment and have a BMI of 16 or below. So our definition of ‘severe anorexia nervosa’ is somewhat more lenient than that used normally in the NHS.

The following questions are specifically about the treatments as delivered in the trial, i.e. using an inpatient treatment or stepped-care day treatment approach as described earlier, and your views around how these treatment pathways might work in the trial.

- What impact do you think the stepped-care day treatment approach can have on patients with severe anorexia nervosa?
- What are your concerns about this treatment pathway?
  - E.g. any challenges, areas of uncertainty
- What benefits do you believe this treatment pathway will have for patients with severe anorexia nervosa? And for their carers?
- How do you think the stepped-care approach might affect your team?
  - E.g. workload, the type of work you do
- Do you think this treatment pathway could have any unintended consequences?

We’ve already spoken a bit about the general positives and negatives of inpatient treatment, but we would now like to hear any additional thoughts about the treatment pathway as delivered in the trial (e.g. for those patients with a BMI of 16 or below). So, for those who are randomly allocated to the inpatient treatment pathway:

- What impact do you think the inpatient treatment approach can have on patients with severe anorexia nervosa? And for their carers?
- What are your concerns about this treatment pathway?
  - E.g. any challenges, areas of uncertainty
- What benefits do you believe this treatment pathway will have on patients with severe anorexia nervosa?
- Do you think this treatment pathway could have any unintended consequences?
- Do you think the trial might have an effect on patients who aren’t in the trial?
- If so, do you think these effects may be different in an inpatient or day patient setting?
- What do you think it will be like for staff to manage patients in an inpatient/day patient setting while some are involved in the trial and others are not?
  - Do you expect this to cause any challenges? Are there any opportunities here too?
- How do you think patients will feel about getting involved in the trial?
  - What positives do you think they might see about participation?
  - What negatives do you think they might see about participation?
- How do you think patients’ families and carers will feel about their loved one participating in the trial?
  - What positives and negatives do you think they will have?
- How do you think the carers will feel about their own participation in the trial?
- What could we do to help you and your team during this trial?
  - E.g. any support, information, training etc.

As I mentioned previously, in the stepped care treatment approach the decision around stepping patients up/down between day- and inpatient treatment will be dependent on risk assessments conducted by the clinical team. The risk assessment will assess medical risk using objective indicators of nutritional status (e.g. BMI, weight change), cardiovascular function (blood pressure, pulse, postural drop), laboratory parameters and other physical risk indicators. In addition, we have added a psychiatric/psychosocial risk category (including e.g., suicidality; major self-harm; availability of support, safe-guarding concerns, patient/carer concerns etc.). This will be conducted by the clinical team on a weekly basis using a simple traffic light system which will help determine the best treatment setting for patients. For instance, patients with any indicators in the red risk category will usually be admitted to or continue inpatient treatment and those with all/predominately green risk indicators can remain/step-down to day patient treatment.

- How do you feel about using a risk assessment tool like this for this decision making?
  - E.g. is a similar tool used in your current work?
- Do you have any concerns about using such a risk assessment tool for decision making around stepping patients up and down?

Thank you. Is there anything else you would like to add that you feel we haven’t covered in this interview, or anything you would like the research team to know?
